# Supplementary material for: Glutamine and Cholesterol Plasma Levels and Clinical Outcomes of Patients with Metastatic Castration-Resistant Prostate Cancer Treated with Taxanes
Source: Cancers (Basel). 2021 Oct 1;13(19):4960. doi: 10.3390/cancers13194960 (PMC8507765; doi:10.3390/cancers13194960)
Supplement: Supplementary file 1 [file cancers-13-04960-s001.zip › cancers-1383327-supplementary.pdf]

SUPPLEMENTARY MATERIAL

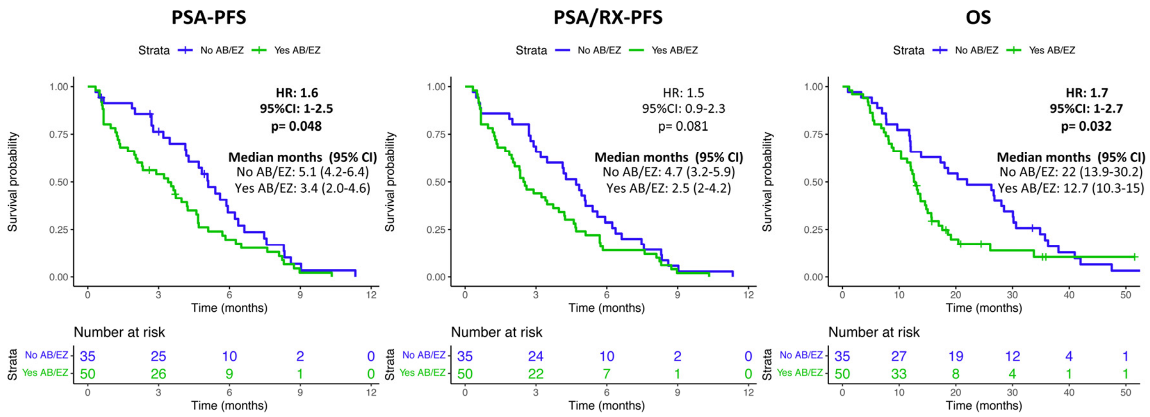

Figure S1 Effect of receiving abiraterone or enzalutamide prior to taxanes in clinical outcome. A) Kaplan-Meier curves representing PSA-PFS, PSA/RX-PFS, and OS according to whether patients received AB or EZ prior to taxanes. CI: confidence interval; HR: hazard ratio; AB: abiraterone; EZ: enzalutamide; Wald test (p).

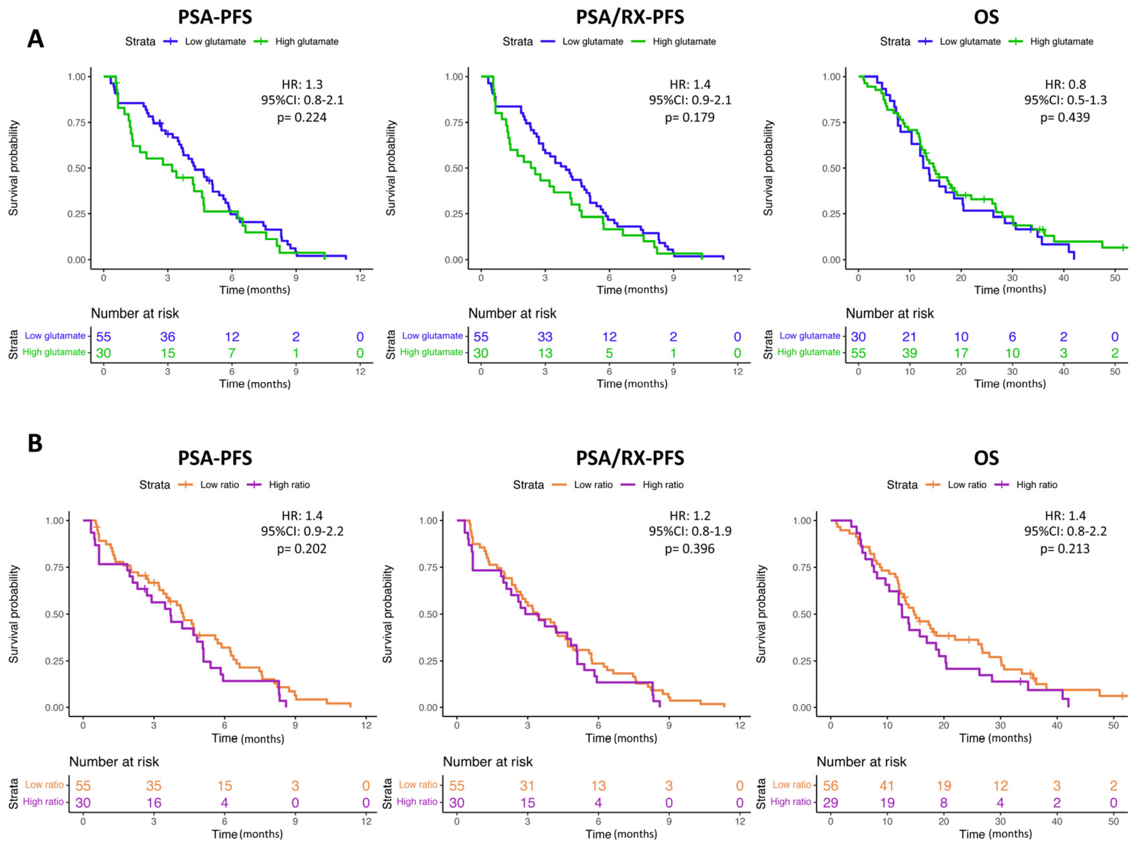

Figure S2 Glutamate and ratio glutamine/glutamate at plasma and clinical outcome. A) Kaplan-Meier curves representing PSA-PFS, PSA/RX-PFS, and OS according to glutamate levels; B) Kaplan-Meier curves representing PSA-PFS, PSA/RX-PFS, and OS according to the ratio glutamine/glutamate levels; CI: confidence interval; HR: hazard ratio; Wald test (p).

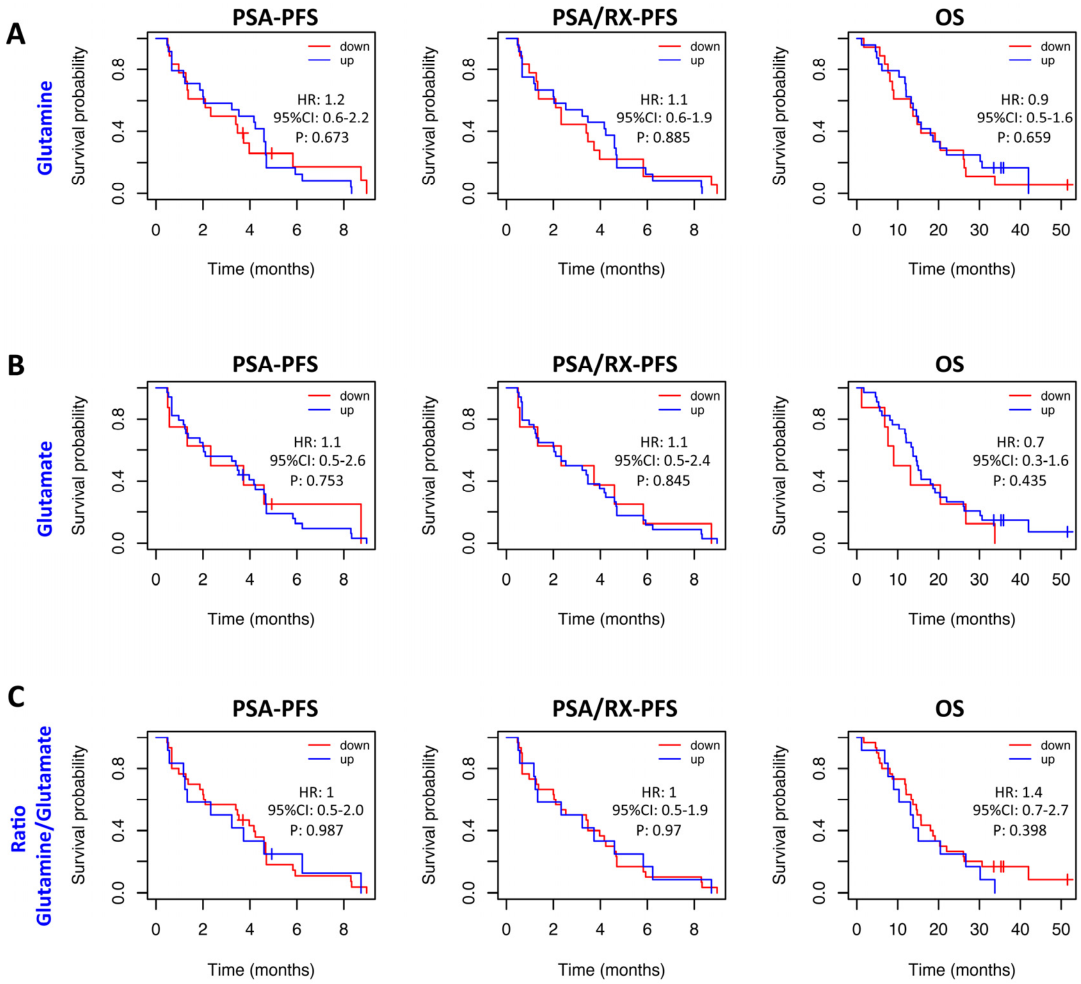

Figure S3 Changes of glutamine, glutamate and ratio glutamine/glutamate levels at plasma before and after taxanes exposure and clinical outcome. Patients whose levels after treatment went up and down were compared. A) Kaplan-Meier curve representing PSA-PFS, PSA/RX-PFS and OS according to changes in glutamine levels; B) Kaplan-Meier curve representing PSA-PFS, PSA/RX-PFS and OS according to changes in glutamate levels; C) Kaplan-Meier curve representing PSA-PFS, PSA/RX-PFS and OS according to changes in the ratio glutamine/glutamate. CI: confidence interval; HR: hazard ratio; Wald test (p).

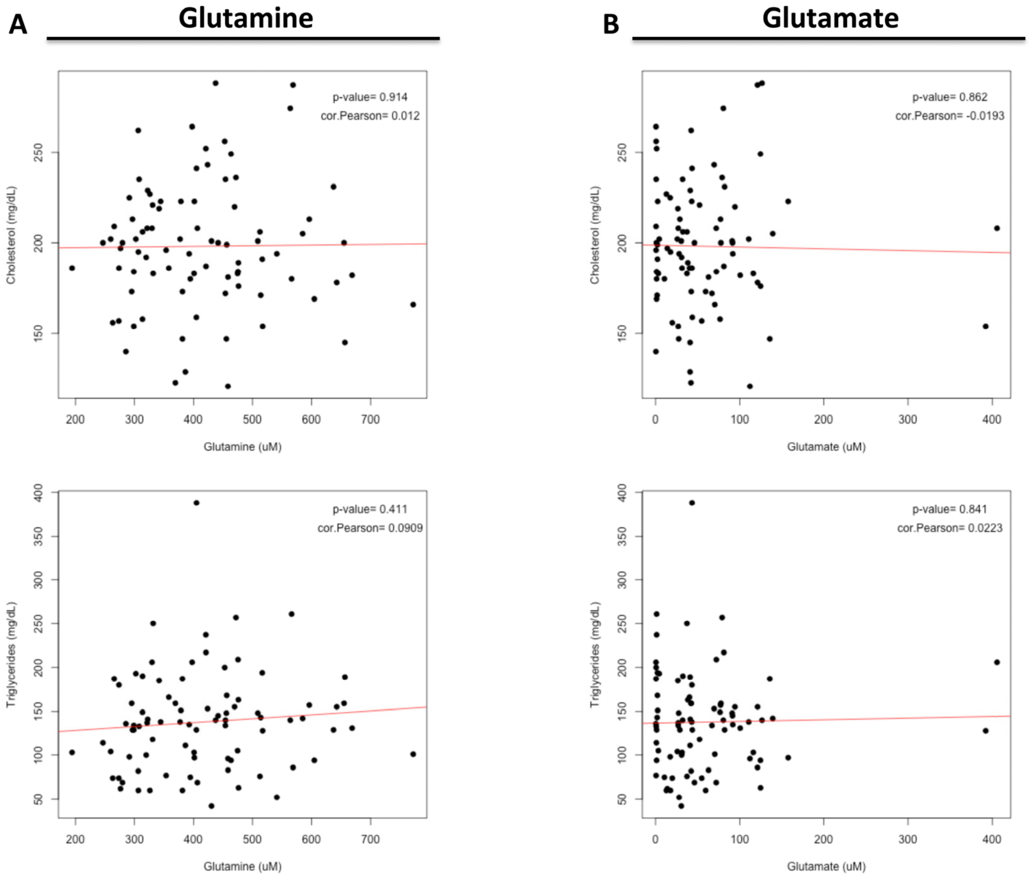

Figure S4 Correlation with lipids. A) Correlation graph between glutamine and cholesterol (up) and triglycerides (down); B) Correlation graph between glutamate and cholesterol (up) and triglycerides (down); Pearson coefficient (cor) and p-values are shown.

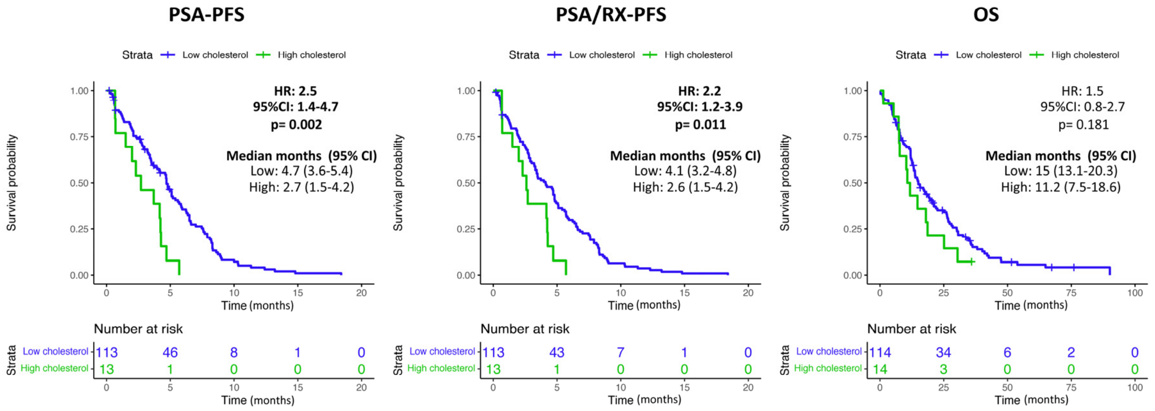

Figure S5 Cholesterol and clinical outcome in the global cohort. A) Kaplan-Meier curves representing PSA-PFS, PSA/RX-PFS, and OS according to cholesterol levels. Cut-off for cholesterol levels was the one used at routinely blood test analysis (247 mg/dL). CI: confidence interval; HR: hazard ratio; Wald test (p).

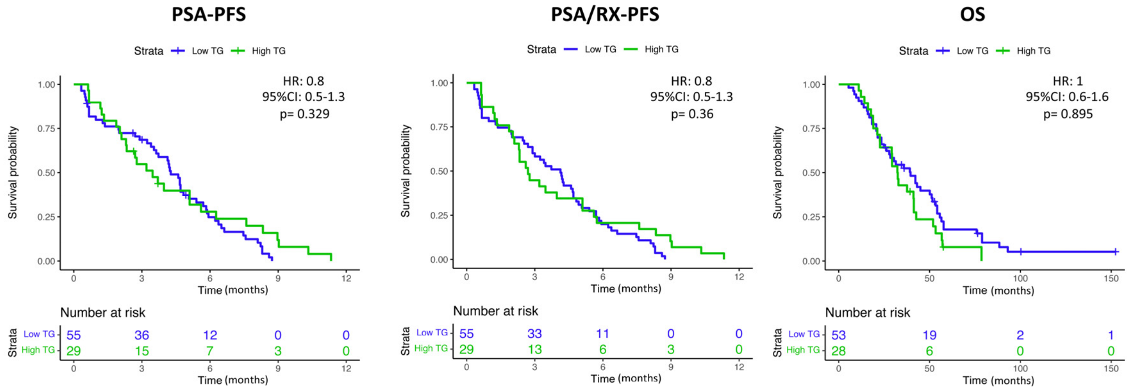

Figure S6 Triglycerides (TG) and clinical outcome. A) Kaplan-Meier curves representing PSA-PFS, PSA/RX-PFS, and OS according to TG levels. CI: confidence interval; HR: hazard ratio; Wald test (p).
